# Food-Derived Compounds Extend the Shelf Life of Frozen Human Milk

**DOI:** 10.3390/foods14122018

**Published:** 2025-06-07

**Authors:** Justin E. Silpe, Karla Damian-Medina, Bonnie L. Bassler

**Affiliations:** 1 Department of Molecular Biology, Princeton University, Princeton, NJ 08544, USA; justin@pumpkin-baby.com; 2 Howard Hughes Medical Institute, Chevy Chase, MD 20815, USA; 3 PumpKin Baby Inc., Princeton, NJ 08540, USA; karla@pumpkin-baby.com

**Keywords:** human milk, milk storage, breast pump, lipolysis, high-throughput screening, milk preservation, freezer storage, breastfeeding, household storage

## Abstract

Breastmilk is known to provide optimal nutrition for infant growth and development. A cross-sectional analysis of nationally representative US data from 2016 to 2021 revealed that >90% of lactating mothers reported using breast pumps to express milk. We conducted a survey of *n* = 1049 lactating or recently lactating individuals from a US nationally representative population to explore breastmilk storage practices among this group. The data revealed that 83% of respondents store breastmilk in their homes, with 68% using freezers to do so for >1 month. The lowest available temperature in most household freezers is −20 °C, a temperature that is inadequate to maintain human milk’s emulsified structure, leading to separation, degradation of fats, loss of key vitamins, and changes in palatability. We developed a first-of-its-kind high-throughput screening platform to identify food-derived compounds and combinations of compounds that, when added to human breastmilk, preserve fat content, retain antioxidant capacity, and reduce production of rancid-associated free fatty acids during extended freezer storage. Our screening identified pectin (0.5% *w*/*v*) and ascorbic acid (100 μg/mL) as optimal preservation agents. Compared to untreated controls, this formulation reduced glycerol production by approximately 60% and maintained antioxidant capacity after 6 months of storage at −20 °C. Lysozyme and protease activity were maintained at >75% of the levels in fresh breastmilk. This formulation represents a lead for the development of safe and affordable frozen breastmilk shelf-life extenders for at-home use to increase the longevity of stored breastmilk.

## 1. Introduction

Human breastmilk is the gold standard for infant nutrition, containing a complex blend of vital nutrients and other factors that cannot be synthesized or sourced from other mammals yet are essential to meet life’s critical early milestones. The World Health Organization (WHO) recommends infants be exclusively breastfed for the first 6 months of life. However, fewer than half of all infants currently meet this guideline [1], and the majority of parents (60%) fail to accomplish their own breastfeeding goals [2]. In today’s work–life, many new parents are full-time employed at the time of childbirth, and most return to their professions while their infants still require breastmilk [3]. Indeed, over 90% of US lactating mothers now report using breast pumps [4], making storage quality a pressing concern. Alarmingly, in the US, 43% of women leave the workforce within 3 months of childbirth [5], and those who remain earn ~30% less in subsequent years than they did the year before the birth of their first child [6]. One reason cited for leaving the workforce is the desire to take care of the infant, for which feeding the baby is one of the highest-ranking needs [7]. This desire often conflicts with the demands of returning to work, as evidenced by the fact that among the 67% of working mothers who initiated breastfeeding in one study, only 10% continued once back to work [8].

Given the critical role breastmilk plays in infant development, many parents rely on freezer storage to enable continued breastfeeding. However, household freezers fail to prevent separation of milk’s emulsified components, and freezing can accelerate the degradation of proteins, vitamins, and other bioactive compounds [9,10,11,12,13,14]. The structural changes induced by freezing can alter milk palatability, potentially leading to infant rejection [15]. In fresh human milk, lipids are encapsulated within milk fat globules (MFGs), which are surrounded by fat globule membranes, termed milk fat globule membranes (MFGMs). MFGMs protect lipids from lipases present at the globule interface and/or in the aqueous phase. The crystallization of milk lipids that occurs during freezing can damage MFGMs, allowing lipases access to lipids. Lipases hydrolyze milk triglycerides into free fatty acids (FFAs) and glycerol. FFAs, particularly volatile short- and intermediate-chain fatty acids, coincide with rancid flavors in milk products [15]. Indeed, FFA production affects flavor and accelerates oxidative processes that further degrade sensory and nutritional milk quality. These issues are magnified by the fact that most home cooling systems are subject to temperature fluctuations and variable personal habits [16], further degrading milk quality. Consequently, parents often face the dilemma of discarding stored milk or feeding their infants breastmilk they suspect is compromised.

Evidence supporting the changes human milk undergoes following freezing includes a study reporting that 25% of infants refused to consume previously frozen human milk, and in 95% of these cases, the milk was described as smelling “off” [17]. In the current work, we refer to off-smelling milk as being rancid. Rancidification is a process in which fats undergo oxidation, autoxidation, hydrolysis, and/or lipolysis upon exposure to air, light, moisture, or enzymes [15,18,19,20]. Rancid milk, while still nutritious and safe to consume [21], is characterized by an unpleasant taste and odor. Many parents are loath to feed rancid milk to their infants, and not all babies accept such milk, suggesting that these changes prevent some infants from receiving any nutritional benefit from breastmilk. Discarding or rejecting breastmilk forces suboptimal feeding regimes for parents (i.e., resorting to commercial formulas despite knowing them to be imperfect substitutes for breastmilk). Compounding this problem is that current methods to facilitate milk storage, such as freeze-drying, pasteurization, and scalding, have limitations, including high costs, loss of nutritional content, and impracticality for home use. Therefore, innovative approaches that can mitigate the adverse effects freezing has on breastmilk, while remaining affordable and accessible are essential. In this work, we sought to understand current breastmilk storage practices and challenges encountered. Based on the data acquired, we propose safe and easy-to-use solutions.

## 2. Materials and Methods

### 2.1. Study Design

This study was conducted in two phases, each independently approved by the Institutional Review Board (IRB) at Princeton University (IRB IDs: 16889 and 15531). Informed consent was obtained from all participants before their inclusion in the study. Phase 1 was focused on a cross-sectional nationwide survey to identify challenges associated with breastmilk storage practices. Phase 2 involved freshly expressed breastmilk collection to evaluate the influence of at-home storage practices on the nutritional quality of human milk.

#### 2.1.1. Phase 1: Data Collection on Breastmilk Storage Practices

The survey was conducted on a research platform generated and managed by Centiment Co (https://www.centiment.co/), which handled the deployment and data collection. Initially, 2013 participants were screened, and 1049 were selected based on specified eligibility criteria identified within Centiment’s database. The participant’s youngest child must have been born between 2020 and 2024; the infant must have received exclusively breastmilk or a combination of formula and breastmilk during the first six months of life. In cases where formula was provided during the first six months, the participant must have attempted to breastfeed and confirmed that information about their breastfeeding experience could be included. The questionnaire was available on the Centiment platform from 30 May to 6 June 2024 and included a series of yes/no and multiple-choice questions regarding demographics, breastmilk storage methods and durations, reasons for freezing breastmilk, and observed changes in milk quality post-storage. Participants could select more than one answer for how they commonly store their breastmilk (e.g., refrigerator, freezer, refrigerator and freezer). Participants were asked about quality changes in refrigerated milk if they reported using a refrigerator but not a freezer. If a participant selected having used a freezer (with or without a refrigerator), they were asked about quality changes in frozen milk. Additional questions addressed disposal of stored milk and challenges encountered. Original survey questions and exclusion criteria are accessible through Zenodo (refer to Data Availability).

#### 2.1.2. Phase 2: Collection of Human Milk Samples

Freshly expressed human milk samples were collected from 14 lactating human donors. Participants were recruited through advertisements in local parenting groups, social media, and via lactation consultants. Eligibility for donating one fluid ounce (29.6 mL) of freshly pumped breastmilk required participants to be healthy breastfeeding mothers aged 18 to 45 years who had delivered a term infant (born at 37 weeks gestation or later) and who had been exclusively breastfeeding for 12 or more weeks. Donors needed to confirm that they owned and could operate a breast pump, had no history of breast surgery, were not using medications known to affect milk production or composition, and had abstained from alcohol consumption for 48 h prior to collection. Each participant provided informed consent and received instruction on proper milk expression and collection techniques to maintain sample integrity. Donors contributed one fluid ounce of milk obtained from a single, full pumping session, transferring this volume into an unused milk collection bag of their choice or a sterile sample collection vessel provided by the study personnel. No specific instructions were provided regarding whether the milk should be expressed from a single breast or both breasts during the session. These samples were collected by study personnel and immediately transported to the laboratory on ice, where they were processed within 2 h of pickup.

### 2.2. Experimental Conditions and Storage Variables

#### 2.2.1. Analytical Procedures

Microplate readers: BioTek Synergy Neo2 Multi-Mode readers (BioTek Instruments, Winooski, VT, USA) with BioStack and BioSpa functionalities were used for lipase, lysozyme, protease, glycerol, FFA, and total antioxidant capacity measurements, as described below. Unless otherwise noted, fresh human milk served as the comparison standard, and water/DMSO was used as the vehicle control. To minimize freeze–thaw issues, data for each time point were obtained using dedicated plates that were thawed only once at the specified analysis time. In the initial screening, all compounds were assayed using milk from a single donor across multiple plates, with each plate containing all controls. In validation experiments, individual plates were prepared for each donor sample, and all plates for a given time point were processed simultaneously to ensure consistent handling conditions.

High-throughput screening (HTS)*:* A multi-step HTS approach was developed to identify compounds that reduce lipolysis in human breastmilk during extended freezer storage. Briefly, 150 μL aliquots of freshly expressed (same-day) human milk collected from a single donor were dispensed into 30 × 96-well plates (Corning 3903, Corning Inc., Corning, NY, USA). Food- and nutrient-specific chemical libraries (MedChemExpress, MedChemExpress LLC, Monmouth Junction, NJ, USA) Food Additive Library, Food Sourced Compound Library, and Biolog (Biolog, Inc., Hayward, CA, USA) Phenotype Microarrays PM1-5) were transferred into milk-containing wells using Scinomix 96-well pin replicators (Scinomix, Earth City, MO, USA). The volume transfer from the pins was empirically determined to be ~1.5 μL, leading to a 1% *v*/*v* treatment. Controls of 1% *v*/*v* DMSO and 1% *v*/*v* water were included in each plate. Plates were foil-sealed and stored at −20 °C. At regular intervals for up to 1 week, plates were thawed and samples measured for lipolysis by transferring 2 μL of each treated milk sample into 96-well plates containing a working solution (98% *v*/*v*, final concentration) of EnzChek Lipase Substrate (Thermo E33955, Thermo Scientific, Waltham, MA, USA). The working solution was prepared by dissolving 100 μg of substrate in 20 μL DMSO, followed by 5000-fold dilution into PBS immediately prior to the experiment. Plates were incubated at 37 °C in the microplate reader for 2–6 h, and Relative Fluorescence Units (λ_ex_/λ_em_ of 500 nm/530 nm) were measured. The method was validated using lipoprotein lipase from bovine milk (at 25 μg/mL with ≥2000 units/mg, Sigma, L2254, Sigma-Aldrich, St. Louis, MO, USA). Hit criteria were derived from Z-scores and defined as wells with RFUs ≤ 400.

Lysozyme: Lysozyme activity was assessed using the EnzCheck Lysozyme Assay Kit (Thermo E22013). A 1 mg/mL stock suspension of DQ lysozyme substrate was prepared by adding 1 mL of deionized water to the lyophilized substrate. The working solution was prepared by diluting the stock suspension to 50 µg/mL in 1× reaction buffer (0.1 M sodium phosphate, 0.1 M NaCl, pH 7.5). Human milk samples (2 µL) were diluted with 48 µL reaction buffer in black 96-well plates (Corning 3904) and mixed with 50 µL of the working solution. Plates were incubated at 37 °C for 30 min, protected from light. Fluorescence intensity was measured at λ_ex_/λ_em_ of 485 nm/530 nm using a microplate reader.

Protease: Protease activity was determined using the EnzCheck Protease Assay Kit (Thermo E6638). A 1 mg/mL stock solution of BODIPY FL casein substrate was prepared by adding 0.2 mL PBS to the lyophilized substrate. The working solution was prepared by diluting the stock solution to 10 µg/mL in 1× digestion buffer. Human milk samples (2 µL) were diluted with 48 µL digestion buffer in black 96-well plates (Corning 3904) and mixed with 50 µL of the working solution. Plates were incubated at 37 °C for 30 min protected from light. Proteolytic cleavage releases fluorescent BODIPY FL-labeled peptides, which were measured at λ_ex_/λ_em_ of 500 nm/535 nm using a microplate reader.

Glycerol: Glycerol content was quantified using the Glycerol-Glo Assay Kit (Promega J3151, Promega Corporation, Madison, WI, USA). Human milk samples (2 µL) were diluted with 48 µL glycerol lysis solution from the kit in white-walled 96-well plates (Corning 3903) and incubated for 30 min at 37 °C. The glycerol detection reagent was prepared by adding 10 µL of reductase substrate per mL of glycerol detection solution and equilibrating for 60 min at room temperature. Kinetic enhancer (10 µL per mL) was then added to the detection reagent. An equal volume (50 µL) of this prepared glycerol detection reagent was added to each well containing the diluted milk samples. Plates were shaken for 30 s and incubated at room temperature for 60 min. Luminescence was measured using a microplate reader.

Total Antioxidant Capacity: Total antioxidant capacity of human milk was assessed using the Total Antioxidant Capacity Assay Kit with protein mask functionality (Abcam ab65329, Abcam, Cambridge, UK). The Cu^2+^ working solution was prepared by diluting Cu^2+^ Reagent 50× with Assay Buffer XXIV. Human milk samples (10 µL) were first diluted 1:1 with the protein mask reagent and then brought to a final volume of 100 µL before adding 100 µL of the Cu^2+^ working solution. The plates were sealed and incubated at room temperature for 90 min on an orbital shaker protected from light prior to measuring absorbance at 570 nm using a microplate reader.

#### 2.2.2. Quantitation and Statistical Analyses

Software used to acquire and analyze data generated in this study consisted of BioTek Gen5 v.3.11 (BioTek Instruments, Winooski, VT, USA) for assessment of lipase, lysozyme, protease, glycerol, free fatty acids (FFA), and total antioxidant capacity. Independent replicates are defined as milk samples obtained from the same donor that were individually prepared, stored in separate tubes, measured in distinct wells, and processed on the same day. Unless otherwise noted, data are presented as the means ± SDs. Where indicated, biological replicates are defined as milk samples obtained from different donors and analyses carried out on the same day. Statistical analyses were conducted to evaluate the effects of various treatments on antioxidant capacity, lipolysis, and glycerol accumulation in stored human milk. One-way ANOVA was used to compare treatment effects on antioxidant capacity and lipolysis. Two-way ANOVA was used to analyze two independent variables: treatment × concentration effects and treatment x donor effects. Repeated measures ANOVA was used for time-course data when the same samples were measured at multiple time points. Chi-square tests were used to analyze categorical survey data. When significant effects were detected, Tukey’s multiple comparisons test was used for post hoc analysis. Homogeneity of variances was evaluated using Bartlett’s and Brown–Forsythe tests. Normality of residuals was verified via inspection of Q-Q plots and calculation of skewness and kurtosis values. Data were considered normally distributed when skewness fell within ±1 and kurtosis within ±2. All datasets met these criteria or exhibited only marginal deviations, for which ANOVA is robust given adequate sample sizes. Full normality test statistics are available in the source data file. A *p*-value of <0.05 was considered statistically significant. For dose–response experiments evaluating lipolysis inhibition by food-derived compounds, repeated-measures ANOVA was performed across a dilution series. Data visualization and analysis were performed using GraphPad Prism version 10 (Dotmatics, Bishop's Stortford, UK).

## 3. Results

### 3.1. Breastmilk Freezer Storage Respondent Demographics and Practices

It is generally understood, particularly in developed countries with limited parental leave policies, that nursing parents depend heavily on storage of expressed breastmilk. However, challenges associated with pumping and the consequences of at-home storage on the nutritional quality of human milk have not been thoroughly quantified. To address this gap, we conducted an anonymous, electronic, retrospective survey to garner data on current breastmilk storage practices, the hurdles faced, and the consequences of the use of pumped human milk as reported by parents. The survey was conducted on a nationally representative sample of 1049 reproductive-age individuals across 50 states who had considered, attempted, or successfully breastfed an infant within the past 3 years (see survey criteria in Supplementary Materials and Figure S1a–f). The survey asked participants to indicate the methods used to feed their infants during the first 6 months of life. Forty-six percent reported feeding their infant exclusively breastmilk, while 11% used only formula, and 44% fed their infants a combination of breastmilk and formula (Figure S1a). Regarding how breastmilk was provided to infants, 55% of respondents reported using a combination of both breastfeeding and pumped milk, 26% reported that breastmilk was delivered exclusively from the breast, and 19% exclusively pumped and bottle-fed breastmilk (Figure S1b). The average age of participants who completed the survey was 30.6 ± 6.7 years old. Sixty-one percent were White, 23% were Black or African American, 17% were Hispanic or Latino, 4% were Asian, 2% were Native American or Alaska Native, and 1% were Middle Eastern or North African. Forty-four percent of participants were full-time employed, 19% were part-time employed, 22% were unemployed, and 4% were full- or part-time students. Approximately 81% of participants reported having an annual household income between USD 20,000 and 199,000/year (Table S1).

The survey responses confirmed that storage is a common practice among parents who use breastmilk. Specifically, 83% of respondents reported having stored breastmilk, of whom 68% reported using a freezer to do so (Table S2). The duration of storage in freezers varied, with 31% of respondents storing milk for less than 1 month, 40% for 1 to 3 months, 17% for 4 to 6 months, and 12% for more than 6 months (Figure S1c). Reasons for freezing breastmilk were related to oversupply (63%), work (38%), and travel (23%) (Table S2). The survey also revealed that surprising amounts of breastmilk go to waste. Specifically, 75% of respondents reported discarding breastmilk, primarily due to incomplete feeding (62%), questionable proper storage (38%), and/or sensory changes (i.e., “the milk smelled off”, 23%) (Figure S1d). Changes in smell or taste following defrosting were reported by nearly 20% of respondents (Table S2). These sensory alterations presumably affect the acceptability of stored milk by infants, as 26% of respondents reported experiencing occasional or persistent infant rejection of the milk (Figure S1e). Moreover, the proportion of respondents reported that changes in the milk increased in a storage-time dependent manner (Figure S1f), suggesting that at least some of the observed alterations are driven by the storage process itself rather than by interindividual differences in breastmilk composition or variability in human practices.

### 3.2. Changes That Occur in Human Milk During Storage

With the goal of improving the shelf life of stored human milk, we devised a high-throughput, small-molecule screening (HTS) approach to identify compounds naturally present in foods that stall the rancidification process. To verify that lipolysis and lipolytic byproducts can be reliably measured in small volumes of human milk, we adapted existing assays for use with human milk. One assay employed a fluorescent fatty acid conjugate consisting of a 4,4-difluoro5-methyl-4-bora-3a,4a-diaza-s-indacene-3-dodecanoic acid (BODIPY) dye and a 4-((4-(dimethylamino)phenyl)azo)benzoic acid (DABCYL) quencher to quantify active lipolysis at the point of testing (BODIPY-FA). Second, a coupled-enzyme assay was used to measure glycerol, a product of triglyceride metabolism, as a proxy for fat breakdown. This assay couples glycerol metabolism to NADH production, which drives a luciferase reaction generating bioluminescence emission proportional to glycerol concentration (Glycerol-Glo-glo). Figure S1g (upper) shows that the rate of lipolysis generally decreased during human milk freezer storage but remained measurable. Moreover, the concentration of glycerol increased across all freezer storage time points (Figure S1g (lower)). These data demonstrate that lipolysis and the accumulation of lipolytic byproducts can be measured as they occur in breastmilk. Our assays are quantitative, scalable (96- and 384-well plate formats), and adapted for low milk volumes (2 μL per condition tested), making the strategy amenable to HTS. HTS is a cornerstone approach used in drug discovery and has broad applications in chemistry and biology, but to our knowledge, it has never been applied to breastmilk.

### 3.3. High-Throughput Screening of Food-Derived Compounds for Extending Stored Human Milk Shelf Life

**Figure 1:**
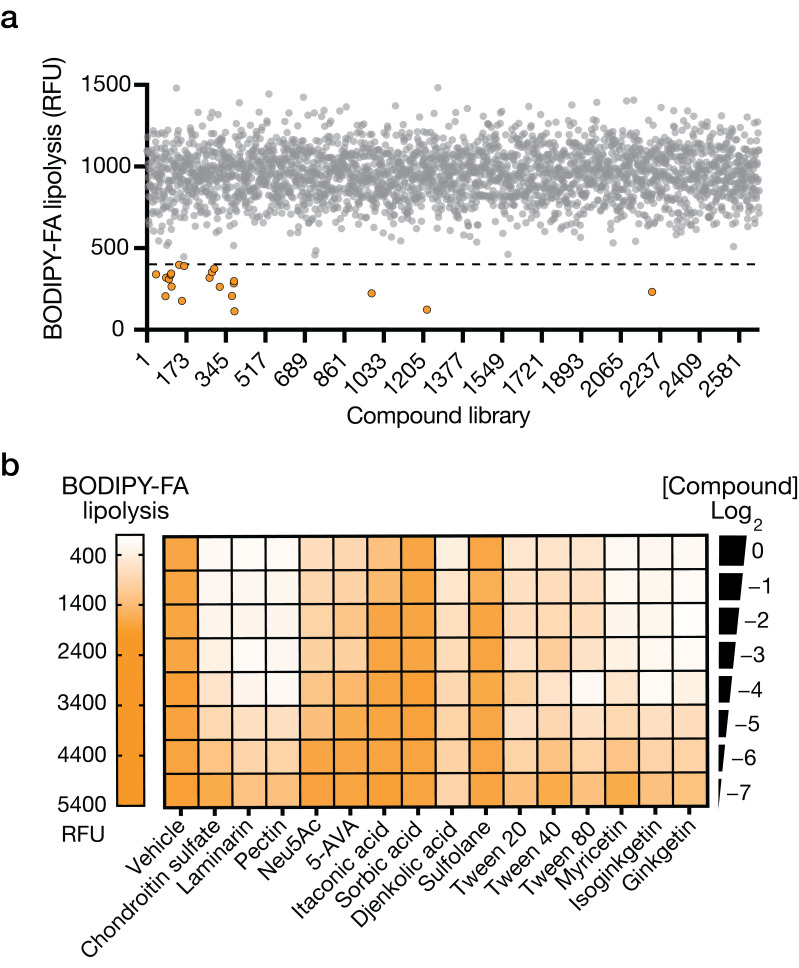
High-throughput screening of food-derived compounds for inhibition of lipolysis in frozen human milk. (**a**) Food-derived compounds screened for breastmilk lipolysis inhibition. Orange dots indicate compounds reducing lipolysis below the threshold (dashed line); gray dots show inactive compounds. Data represent the initial HTS readouts. (**b**) Validation of selected hit compounds from panel (**a**). The heatmap displays lipolysis inhibition in relative fluorescent units (RFU) at different compound concentrations. Compound concentrations are expressed as dilutions (Log_2_(0) to Log_2_(−7)) of the screening library stock solutions. Ne5Ac: N-Acetylneuraminic acid; 5-AVA: 5-Aminovaleric acid. Significant differences were observed across treatments and time points (*p* < 0.0001), indicating that these compounds influence milk lipolysis dynamics during freezer storage. Data represent the median of three independent replicates.

We screened approximately 2750 food-derived compounds from three libraries (Biolog Phenotype Microarrays, MCE Food Additive Library, and MCE Food-Source Compound Library) for the ability to attenuate lipolytic activity, as measured by BODIPY-FA, after 1 week of household −20 °C freezer storage. Our screening process produced 21 hits, defined as yielding <400 relative (RFU) fluorescent units from the BODIPY-FA probe (Figure 1a), 15 of which repeated upon retest. A list of the validated hits from our high-throughput screening, including their chemical classifications and relative efficacies, is provided in Table S3. We assessed the 15 candidate hits for their abilities to reduce lipolysis and FFA accumulation after 3 months of freezer storage (Figure 1b and Figure S1h, left, respectively). Importantly, we demanded that the compounds not suppress lysozyme and protease activity, two important, non-fat-related enzyme activities known to be present in human milk (Figure S1h, middle and right, respectively). We defined lysozyme and protease retention as >75% of the levels in fresh or untreated breastmilk. Using these criteria, we identified nonionic surfactants, glycosaminoglycans, pectins, and specific biflavonoids as hits of interest. Based on these data and, as proposed in our model (see Section 4), we hypothesize that the compounds slow the degradation of breastmilk by stabilizing its structural integrity without inhibiting important enzymes that the infant requires upon ingestion.

**Figure 2:**
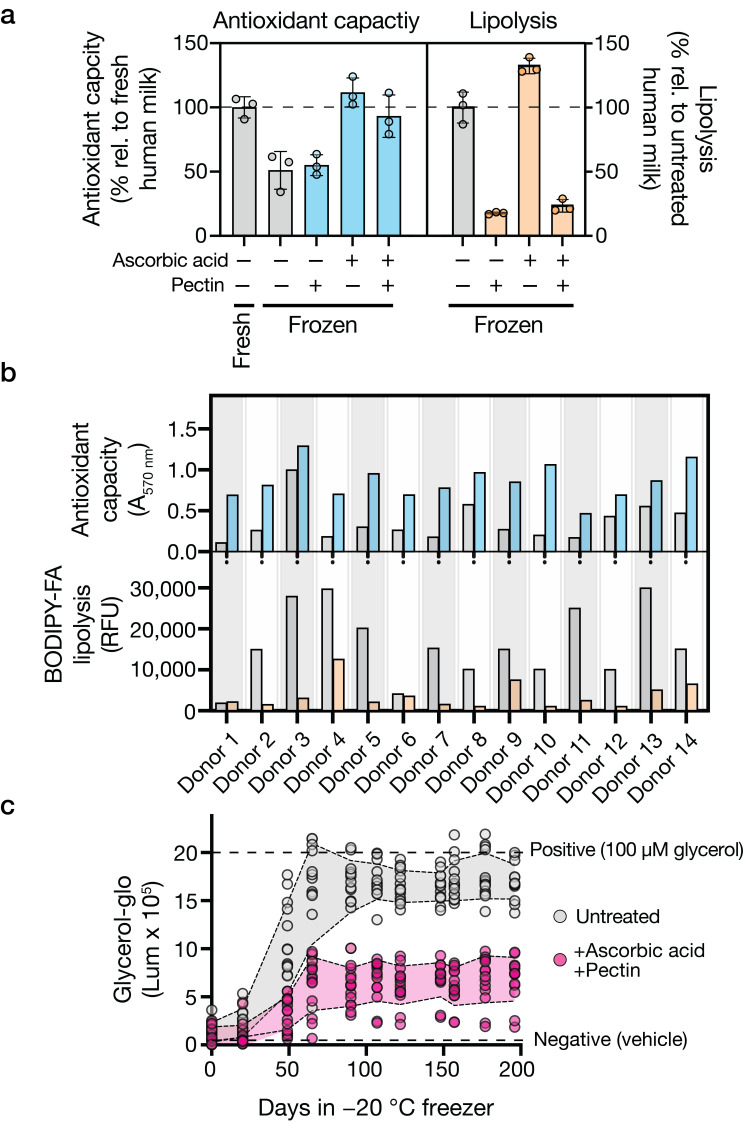
Effects of pectin and ascorbic acid on antioxidant capacity, lipolysis, and glycerol accumulation in frozen human milk. (**a**) Antioxidant capacity and lipolysis in milk treated with 100 μg/mL ascorbic acid and/or 0.5% *w/v* pectin. No treatment (–) or the designated treatment (+) is indicated under the *x*-axis. Values are normalized to untreated fresh milk (antioxidant capacity) or untreated frozen milk (lipolysis). Data show means ± SDs of three independent replicates. Treatments significantly influenced antioxidant capacity (*p* = 0.0003) and lipolysis (*p* < 0.0001). Gray bars: untreated samples; colored bars: treated samples (blue: antioxidant capacity; orange: lipolysis). (**b**) Donor variation (*n* = 14) in antioxidant capacity (A_570_ nm, blue) and lipolysis (BODIPY-FA RFU, orange) with and without the combined 100 μg/mL ascorbic acid and 0.5% *w/v* pectin treatment. Individual donor values are shown to illustrate inter-individual variability. Data show individual donor measurements. Statistical analysis revealed a significant effect of treatment on the measured outcomes (*p* < 0.0001). Gray: untreated; colored: treated samples. (**c**) Glycerol accumulation during −20 °C storage of human milk with and without the combined 100 μg/mL ascorbic acid and 0.5% *w/v* pectin treatment. Dashed lines indicate positive (100 μM glycerol) and negative (vehicle) controls. Data show means ± SDs of 14 biological replicates. Treatment across groups had a significant effect, with *p* < 0.0001, indicating that the intervention reduced glycerol accumulation compared to controls.

In human milk, endogenous ascorbic acid (i.e., vitamin C) levels reportedly decrease by 20% after 24 h of refrigeration [22], with ascorbic acid declining to undetectable levels in some samples after 2 months of freezer storage [23,24]. Supplementation of human milk with exogenous ascorbic acid (100 μg/mL) preserves antioxidant capacity following 1 month of household −20 °C freezer storage (Figure 2a). To test whether the same effect occurs in the presence of lipolysis-suppressing food-derived compounds, we supplemented human milk with ascorbic acid and hit compounds from the screen. Figure 2a shows that, after 1 month of household −20 °C freezer storage, we achieve additive effects on preservation across combinations. Specifically, ascorbic acid (100 μg/mL) addition did not slow lipolysis, yet it maintained antioxidant capacity. Conversely, a compound identified from our lipolysis-inhibiting screen, pectin (0.5% *w*/*v*), had a limited effect on antioxidant maintenance but effectively curbed lipolysis (Figure 2a). We prioritized pectin for further investigation given its extensive clinical history as a safe dietary product for toddlers and children [25,26,27]. Our findings suggest that combining compounds that influence distinct properties of the milk can maintain multiple qualities related to breastmilk nutritional value and thereby significantly improve breastmilk preservation.

The exact chemical composition of expressed human milk is known to be person-specific and, within a single individual, varies over time, making it difficult to predict if or when stored expressed human milk will become rancid. To test the generalizability of our approach, we expanded our sample size to 14 donors who provided freshly expressed milk. Figure 2b demonstrates that despite wide inter-individual variability in baseline lipolysis activity, the addition of 100 μg/mL ascorbic acid and 0.5% *w/v* pectin prior to freezing proved effective in reducing fat breakdown and increasing antioxidant capacity across donors following 3 months of freezer storage. Moreover, across donors, the combination of ascorbic acid and pectin eliminated, on average, approximately 60% of the glycerol production that normally occurs in frozen breastmilk after more than 6 months of freezer storage (Figure 2c).

## 4. Discussion

### 4.1. Implications of Freezer Storage on Breastmilk Quality, Innovative Approaches to Breastmilk Preservation, and Study Limitations

Most household freezers cool to −20 °C, a temperature that does not adequately preserve the complex emulsified structure of human milk. Moreover, while −20 °C is generally the lowest setpoint for home freezers, a recent report on at-home refrigerated insulin indicates that, in practice, domestic cold-storage conditions rarely meet recommended temperatures (78.8% of refrigerated insulin fell outside of range at least once, with the average insulin being out of range for 3 h per day) [16]. Consistent with this finding, it has been reported that storing human milk under suboptimal freezer conditions can lead to significant alterations in its nutritional composition. Specifically, essential macronutrients such as proteins, carbohydrates, and lipids undergo accelerated degradation. Lipids are particularly affected, displaying a reduction of approximately 3.9% after 7 days and 9% after 3 months of storage [12,28]. Key micronutrients, including vitamins, are also negatively affected following freezing. Lastly, prolonged freezer storage causes milk’s endogenous bactericidal properties to decline [14,29,30,31,32,33,34].

**Figure 3:**
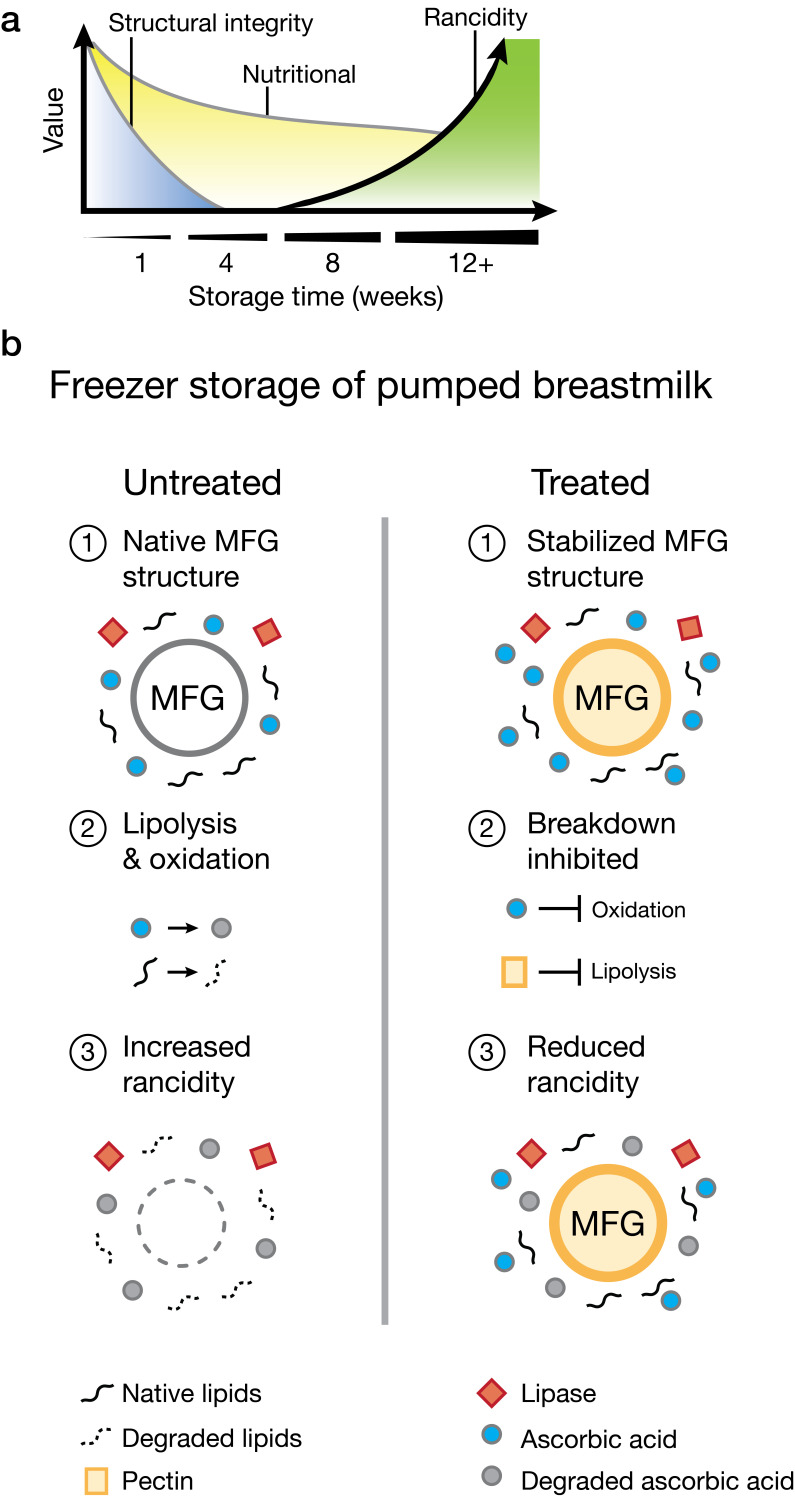
Changes in breastmilk quality during storage and the protective effects of ascorbic acid and pectin treatment. (**a**) Model depicting changes in human milk quality over storage time. At 1 week, degradation begins, leading to a decline in both nutritional value and structural integrity. Between 8 and 12 weeks, rancidification accelerates, marked by an increase in FFAs and other breakdown products, which further reduce nutritional quality and structural integrity. Rancidity continues to rise beyond 12 weeks. (**b**) Schematic model illustrating biochemical and structural changes that occur in human milk during storage. Untreated milk, left: Storage compromises MFG structure, enabling lipase-mediated fat hydrolysis and degradation. Lipolysis of triglycerides and lipid oxidation produces secondary and tertiary products that cause rancidity. Treated milk, right (model proposed in the current work): Pectin preserves MFG structural integrity, limiting lipase access to MFGs. Ascorbic acid provides protection from oxidation. Together, these two mechanisms suppress rancidification.

Germane to the current report, human milk contains antioxidants, including enzymes, vitamins, and non-enzymatic proteins, whose concentrations are affected by storage conditions [35,36,37]. Studies on human milk have shown that antioxidant capacity decreases during both refrigeration and freezing, with freezing causing a larger reduction [38]. Losses in antioxidant capacity are directly related to rancidification: FFAs released from lipolysis are highly susceptible to oxidation, leading to the formation of lipid peroxides and secondary oxidation products, such as aldehydes and ketones, that impart off-flavors and odors [39,40]. The slow freezing rate and variable temperatures of household freezers, and particularly the freeze–thaw process, are known to be damaging, especially to emulsified foods, because of differences in the freezing and melting points of their fat and water phases. Consequently, in the context of breastmilk, the freeze–thaw process drives phase separation, which destroys milk’s natural emulsified structure [41,42]. Phase separation promotes non-specific enzymatic activities, changes in substrate availabilities, and production of reactive intermediates at concentrations that vastly differ or do not occur under physiologically relevant conditions (37 °C) [43]. We propose that losses in structural integrity and changes in constituent composition combine to drive breastmilk rancidification (Figure 3a).

To address the issue of rancidification, we suggest exploiting food-derived compounds to stabilize the structural integrity of breastmilk during freezer storage (Figure 3b). Our hypothesis centers on the protective roles of ascorbic acid and pectin, compounds identified in this work, to preserve the structure and nutritional quality of human milk by stabilizing the emulsion and, in so doing, inhibiting oxidation. Fats in human milk are contained within MFGs, delicate fluid structures enveloped by membranes derived from mammary epithelial cells from the mother’s mammary gland (i.e., MFGMs). As reaction sites, MFGs and MFGMs carry extremely high local concentrations of fats. During storage, particularly under freezing conditions, the structure of the MFGM is compromised [44]. As shown in Figure 3b, freezer storage enables lipases (red diamonds) to access and hydrolyze fats, releasing FFAs (black squiggles). Oxidation of unsaturated fatty acids leads to the formation of primary products, including peroxides, which degrade into secondary products such as aldehydes and unsaturated alkenals. These compounds further react to form tertiary degradation products, including aldol condensation products, hydroxy alkenals, and alkyl furans, contributing to rancidity [45]. We propose that pectin interacts with MFGMs, as has been shown for lipid droplets [46], by forming a surrounding stabilizing layer via surface activity or electrostatic interactions with ionized carboxylic groups that prevent MFG coalescence and enhance emulsion stability (Figure 3b). Regarding oxidation, considering both safety and historical use in infants, ascorbic acid is an ideal antioxidant that is naturally present in all freshly expressed human milk and is an ingredient in commercial infant formulas. While ascorbic acid is a commonly used food additive, it is known to degrade rapidly in a storage-dependent manner [23]. Indeed, to account for expected losses, commercial infant formula- and first food-makers routinely include significant overages of vitamin C. For example, a formula labeled with 8 mg/100 kcal vitamin C may actually contain >400% of that amount, depending on when it is measured [47]. In our system, the addition of ascorbic acid presumably extends breastmilk shelf life by increasing the amount and longevity of antioxidant capacity, which combats FFA oxidation.

A significant attribute of our method is its reliance on naturally occurring compounds rather than potent small-molecule inhibitors that disrupt enzyme activity, such as orlistat, a broad-spectrum lipase inhibitor [48]. By avoiding such inhibitors, we aim to preserve the milk’s natural enzymatic processes and ensure that the nutritional integrity and digestibility of breastmilk remain intact.

Our study has several limitations. Regarding our survey data, while distributed across all US states, the sample exhibits demographic variability in age (SD ± 6.7 years), parity (not specifically controlled), and socioeconomic status (household incomes ranging from USD 20,000 to 199,000), with potential self-reporting biases. Additionally, our survey relied on parents’ subjective assessments of sensory properties of their stored milk and infant acceptance of that milk. This approach may not fully distinguish between infant rejection specifically due to milk quality changes versus general bottle-feeding challenges. Future survey research would benefit from in-depth comparisons of infant feeding preferences and stratified analyses examining how maternal age, lactation experience, cultural background, and socioeconomic characteristics affect milk storage practices and challenges. Regarding the experimental work, the donor pool was relatively small. Moreover, milk sampling techniques were not standardized across donors, which, while potentially limiting, also reflect the variety of real-world practices employed by lactating individuals. Future studies could examine whether the effectiveness of the preservative formulation depends on the specific sampling strategy employed. Despite these limitations, our findings demonstrate the potential of our formulation to preserve nutritional value and reduce fat breakdown in freezer-stored breastmilk. We are currently evaluating the benefits of our formulation on other high-value biological components present in frozen-stored human milk, and, if warranted, we will refine the ingredients to support preservation of additional milk components. Future research will aim to validate our findings through larger, more diverse cohorts and longer-term studies to verify the efficacy and safety of the proposed preservative formulations.

### 4.2. The Role of Breastfeeding in Health and Economic Outcomes

Breastfeeding greatly influences health and prosperity yet remains understudied and undervalued, which negatively affects parents’ abilities—particularly women and mothers—to equitably participate in the workforce and the greater economy. At present, infant formula is included in the WHO calculations of global GDP, but breastfeeding is not, which devalues the contributions it has on society [49]. The WHO has begun the process of quantifying the “work” of breastfeeding for global GDP [50]. Exclusive breastfeeding is estimated to take 1800 h per year of a lactating person’s time, compared to a “full-time job”, which is estimated at 1960 h per year [51]. Given the time invested, it is alarming that the majority of lactating parents (75% in our survey) discard expressed breastmilk and/or face unsolved hurdles to feeding it to infants after storage (26% in our survey).

While improvements in workplace protection policies and advanced pump technologies continue to reduce barriers associated with the expression of human milk, our work highlights that the nutritional quality of expressed breastmilk, and the changes breastmilk undergoes during storage remain unaddressed. Our research shows that the addition of food-derived compounds to breastmilk prior to freezing can delay the rancidification process. Moreover, compounds with multiple modes of action can be combined to inhibit fat breakdown and increase antioxidant capacity in breastmilk, maintaining milk quality during frozen storage for over 6 months. This technology has the potential to promote continued breastfeeding by addressing the unmet need for retaining breastmilk’s nutritional quality during storage. A prevailing theory concerning at-home breastmilk storage attributes rancidity to genetic variability in lipase levels [52]. While we did not specifically test the lipase variation hypothesis in this study, our findings suggest that factors beyond lipase levels, such as MFG damage, phase separation, and antioxidant capacity, affect breastmilk quality during storage.

### 4.3. Regulatory Considerations for Breastmilk Additives

Several classes of compounds with protective effects were among the 15 validated hits: (1) nonionic surfactants including polysorbates; (2) glycosaminoglycans such as N-acetylneuraminic acid and chondroitin sulfate; (3) pectins; (4) biflavonoids including ginkgetin and isoginkgetin; and (5) amino acid derivatives. While these compounds demonstrated efficacy in reducing lipolysis, we prioritized pectin and ascorbic acid for investigation based on their well-established safety profiles in infants, widespread availability, low cost, and complementary mechanisms of action. Future studies will explore additional compounds identified in our screen.

We note that none of the compounds examined in the current work have been evaluated by a regulatory body for use in this application, underscoring the need for further research and development. More generally, no specific regulations exist that govern additives for human milk preservation in home settings. Human milk banking guidelines address pasteurization and storage conditions but do not currently include provisions for additives to extend shelf life. The compounds identified in our study, particularly pectin and ascorbic acid, have well-established safety profiles for food applications and are widely used ingredients in many food products. Pectin has been evaluated by the European Food Safety Authority (EFSA) and is included in foods for infants and young children and in anti-reflux formulas for infants used from birth onward [26]. Ascorbic acid is naturally present in human milk and is a standard ingredient used in infant formulas, typically provided at levels ranging from 6 to 12 mg/100 kcal [47]. Prior to any commercial application, additional safety assessments focusing on the proposed use case are required.

## 5. Conclusions

Here, we made several key findings: (1) a significant proportion of breastfeeding parents (83%) store breastmilk, with 68% using freezers for this purpose; (2) nearly 30% of respondents reported sensory changes in stored milk, and 26% experienced infant rejection of stored milk; (3) our high-throughput screening identified food-derived compounds that preserve breastmilk quality during freezer storage; and (4) a combination of pectin (0.5% *w*/*v*) and ascorbic acid (100 μg/mL) reduced fat breakdown, maintained antioxidant capacity, and eliminated approximately 60% of glycerol production in frozen breastmilk stored for up to 6 months.

The technology we developed leverages safe, food-derived compounds to maintain milk’s structural and nutritional integrity during freezer storage. By extending the usable shelf life of frozen breastmilk, our approach could help parents overcome storage-related challenges and support continued breastfeeding, particularly for parents balancing work and family responsibilities. Future research will focus on optimizing formulations and conducting larger-scale validation studies across more diverse populations.

## Data Availability

Data reported in this study are provided as a source data file. All materials associated with this study have been deposited on Zenodo (doi: 10.5281/zenodo.14252924). Other experimental data that support the findings of this study are available without restriction by request from the corresponding author.

## Supplementary Materials

The following supporting information can be downloaded at https://www.mdpi.com/article/10.3390/foods14122018/s1. Figure S1: Survey results on breastmilk storage practices and biochemical analysis of storage-related degradation. Table S1: Demographic characteristics of US breastfeeding survey respondents. Table S2: Breastmilk storage methods, duration, and reported sensory changes. Table S3: Validated hits from high-throughput screening for breastmilk preservation.

## Abbreviations

5-AVA	5-aminovaleric acid
BODIPY	4,4-difluoro-5-methyl-4-bora-3a,4a-diaza-s-indacene-3-dodecanoic acid
BODIPY-FA	BODIPY–fatty acid conjugate
CDC	Centers for Disease Control and Prevention
DABCYL	4-((4-(dimethylamino)phenyl)azo)benzoic acid
DMSO	dimethyl sulfoxide
FA	fatty acid
FFA	free fatty acid
GDP	gross domestic product
HTS	high-throughput screening
IRB	Institutional Review Board
MFG	milk fat globule
MFGM	milk fat globule membrane
Ne5Ac	N-acetylneuraminic acid
PBS	phosphate-buffered saline
RFU	relative fluorescence unit
US	United States
USD	United States dollar
WHO	World Health Organization
