# Supplementary material for: Food-Derived Compounds Extend the Shelf Life of Frozen Human Milk
Source: Foods. 2025 Jun 7;14(12):2018. doi: 10.3390/foods14122018 (PMC12191995; doi:10.3390/foods14122018)
Supplement: Supplementary file 1 [file foods-14-02018-s001.zip › foods-3633528-supplementary.pdf]

foods

*Supplemental Information*

# Food-Derived Compounds Extend the Shelf-Life of Frozen Human Milk

**Justin E. Silpe [1,2,3], Karla Damian-Medina [3] and Bonnie L. Bassler [1,2,*]**

1 Department of Molecular Biology, Princeton University, Princeton, New Jersey, USA
2 Howard Hughes Medical Institute, Chevy Chase, Maryland, USA
3 PumpKin Baby Inc., Princeton, New Jersey, USA
* Correspondence: bbassler@princeton.edu

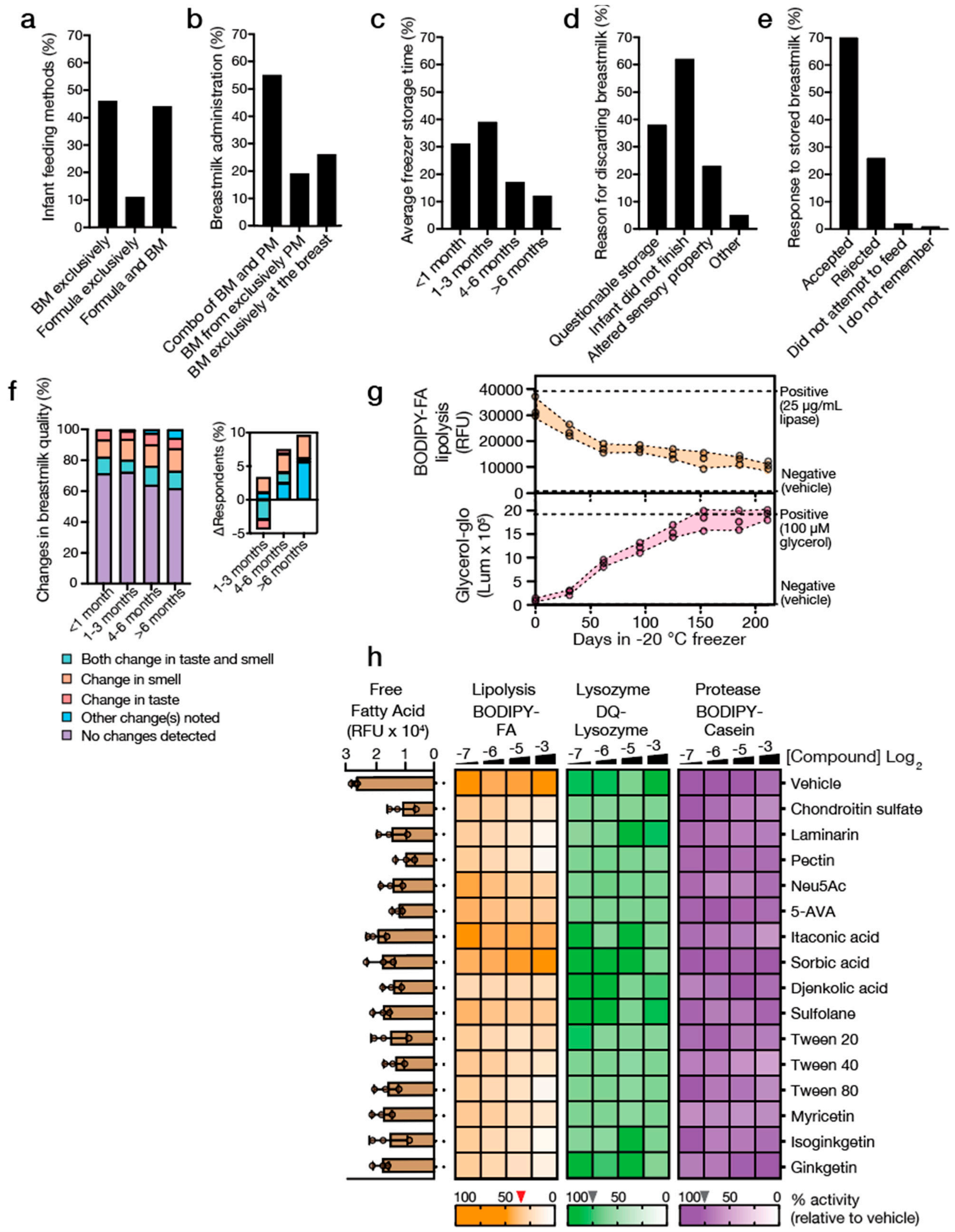


**Figure S1.** Survey results on breastmilk storage practices and biochemical analysis of storage-related degradation.

**(a-f): Survey results from 1,049 respondents on milk storage practices. (a)** Infant feeding methods in first 6 months: exclusive breastmilk ($n$ = 479), exclusive formula ($n$ = 111), or combined ($n$ = 459). A chi-square goodness-of-fit test showed a significant difference in the distribution of feeding methods ($p$ < 0.0001). **(b)** Breastmilk administration: combined

breastfeeding and pumped milk (*n* = 514), exclusive breastfeeding (*n* = 241), or pumped milk only (*n* = 178). BM: breastfed breastmilk; PM: pumped breastmilk. A significant difference in the distribution of feeding methods was observed ($p < 0.0001$). **(c)** Freezer storage duration: <1 month (*n* = 224), 1-3 months (*n* = 283), 4-6 months (*n* = 122), >6 months (*n* = 89). The distribution of storage durations was significantly different ($p < 0.0001$). **(d)** Milk discard reasons: storage concerns (*n* = 293), incomplete feeding (*n* = 486), sensory changes (*n* = 180). The distribution of reasons varied significantly ($p < 0.0001$). **(e)** Infant acceptance of frozen milk: accepted (*n* = 701), rejected (*n* = 269, inclusive of occasional and outright rejection), not attempted (*n* = 17), no recall (*n* = 15). The distribution across response categories was significantly different ($p < 0.0001$). **(f)** Quality changes over storage time, with the inset showing the percent change from the earlier time point. **(g)** Storage-dependent changes at -20 °C: BODIPY-FA lipolysis activity (upper) and glycerol accumulation (lower). Dashed lines: controls (positive: 25 μg/mL lipase or 100 μM glycerol; negative: vehicle). Both markers showed significant changes over time ($p > 0.0001$). **(h)** Validation of HTS hits at a single concentration ($Log_2(-3)$) on FFA levels and, in a concentration-dependent manner, on lipolysis, lysozyme, and protease activities. Compound concentrations and abbreviations as in Figure 1b. Red triangle: normalized lipolysis hit threshold; gray triangles: normalized lysozyme and protease counter thresholds, each relative to vehicle-treated milk. Data are shown as means ± SD (g, h-bars) or median of triplicates (h-heatmaps).

**Table S1.** Demographic characteristics of US breastfeeding survey respondents.

| | **Mean ± SD** |
|---|---|
| **Age** | 30.6 ± 6.7 |
| **Race** | **% (*n*)** |
| White | 61% (644) |
| Black or African American | 23% (239) |
| Hispanic or Latino | 17% (176) |
| Asian | 4% (44) |
| American Indian or Alaska Native | 2% (24) |
| Native Hawaiian or Pacific Islander | 1% (12) |
| Middle Eastern or North African (MENA) | 1% (7) |
| Prefer not to answer | <1% (3) |
| Other | 1% (10) |
| **Employment Status** | **% (*n*)** |
| Full-time employed | 44% (438) |
| Unemployed | 22% (222) |
| Part-time employed | 19% (187) |
| Student (independent of other employment) | 4% (43) |
| Prefer not to answer | 2% (23) |
| Retired | 0.3% (3) |
| Other | 8% (78) |
| **Household Income** | **% (*n*)** |
| $0-$19,999 | 14% (n) |
| $20,000-$49,999 | 34% (n) |
| $50,000-$99,999 | 31% (324) |
| $100,000-$199,999 | 16% (164) |
| $200,000+ | 4% (47) |
| Not sure | 0% (5) |
| Prefer not to answer | 1% (8) |
| **Infant Birth Year** | **% (*n*)** |
| 2020 | 16.9% (177) |
| 2021 | 18% (184) |
| 2022 | 25% (158) |
| 2023 | 27% (284) |
| 2024 | 14% (146) |

**Table S2.** Breastmilk storage methods, duration, and reported sensory changes.

| **Ever stored expressed breastmilk** | **% (*n*)** |
|---|---|
| Yes | 100% (1049) |
| No | 0% |
| **Amount of expressed breastmilk stored** | **% (*n*)** |
| Some (25%) | 39.0% (410) |
| About half (50%) | 41% (425) |
| Most (75%) | 13% (134) |
| All (100%) | 6% (63) |
| I don't remember | 2% (17) |
| **Storage methods of expressed breastmilk** | **% (*n*)** |
| Room temperature | 10% (108) |
| Refrigerator | 64% (673) |
| Freezer | 68% (718) |
| Unsure | 0.2% (3) |
| Other | 0.1% (2) |
| **Reason for freezing expressed breastmilk** | **% (*n*)** |
| Work | 38% (271) |
| Travel | 23% (162) |
| Oversupply | 63% (451) |
| Daycare | 24% (175) |
| Other | 9% (66) |
| **Average storage time of breastmilk in the freezer** | **% (*n*)** |
| Less than 1 moth | 31.2% (224) |
| 1-3 months | 39% (283) |
| 4-6 months | 17% (122) |
| More than 6 months | 12% (89) |
| **Changes in breastmilk quality after defrosting** | **% (*n*)** |
| Change in smell | 13% (93) |
| Change in taste | 6% (45) |
| Change in taste and smell | 10% (71) |
| No changes detected | 69% (498) |
| Other | 2% (11) |
| **Average storage time of breastmilk in the refrigerator** | **% (*n*)** |
| Less than 24 hours | 56% (168) |
| 1 to 3 days | 40% (121) |
| 3 to 5 days | 3% (9) |
| More than 5 days | 1% (3) |
| **Changes in breastmilk quality after refrigerator storage** | **% (*n*)** |
| Change in smell | 14% (41) |
| Change in taste | 7% (21) |
| Change in taste and smell | 10% (30) |
| No changes detected | 67% (203) |
| Other | 2% (6) |
| **Infant response to stored breastmilk** | **% (*n*)** |
| Accepted (same as fresh milk) | 70% (701) |
| Sometimes rejected (or accepted but with more difficulty than fresh milk) | 23% (235) |
| Rejected | 3% (34) |

| | |
|---|---|
| Did not attempt to feed | 2% (17) |
| I do not remember | 1% (15) |
| **Reasons for discarding breastmilk** | **% (*n*)** |
| Questionable storage method (e.g. power outage) | 38% (293) |
| Infant did not finish the feeding | 62% (486) |
| The milk smelled off | 23% (180) |
| Other | 5% (40) |

**Table S3.** Validated hits from high-throughput screening for breastmilk preservation.

| Compound | Chemical Classification | Lipolysis Activity (Inhibition) |
|---|---|---|
| Chondroitin sulfate | Glycosaminoglycan | +++ (71.2%) |
| Laminarin | Polysaccharide | +++ (80.4%) |
| Pectin | Dietary Fiber/Polysaccharide | +++ (80.1%) |
| Neu5Ac | Sialic Acid | ++ (56.9%) |
| 5-AVA | Amino Acid Derivative | ++ (46.3%) |
| Itaconic Acid | Organic Acid | + (14.8%) |
| Sorbic Acid | Organic Acid | – (-9.3%) |
| Djenkolic Acid | Amino Acid Derivative | +++ (76.7%) |
| Sulfolane | Organosulfur Compound | ++ (44.1%) |
| Tween 20 | Nonionic Surfactant | +++ (75.2%) |
| Tween 40 | Nonionic Surfactant | +++ (74.1%) |
| Tween 80 | Nonionic Surfactant | +++ (77.7%) |
| Myricetin | Flavonoid | +++ (71.4%) |
| Isoginkgetin | Biflavonoid | +++ (80.2%) |
| Ginkgetin | Biflavonoid | +++ (76.6%) |

[1] Lipolysis inhibition averaged across $Log_2$(-7, -6, -5, -3) concentrations: +++ = strong inhibition (>60% reduction); ++ = moderate inhibition (30-60% reduction); + = mild inhibition (10-30% reduction); – = minimal inhibition (<10% reduction).
